# Professional development is the key to securing a future rheumatology workforce. Long term evaluation of a summer school for medical students—a national scientific society’s educational initiative

**DOI:** 10.3389/fmed.2024.1413544

**Published:** 2024-09-03

**Authors:** Judith Sautner, Rudolf Puchner, Myriam Reisch, Alois Alkin, Christina Duftner, Christian Dejaco

**Affiliations:** ^1^Department of Medicine II, Lower Austrian Centre for Rheumatology, Karl Landsteiner Institute for Clinical Rheumatology, State Hospital Korneuburg-Stockerau, Stockerau, Austria; ^2^Austrian Society for Rheumatology and Rehabilitation (ÖGR), Stockerau, Austria; ^3^Danube Private University, Krems, Austria; ^4^Department of Rheumatology and Immunology, Medical University Graz, Graz, Austria; ^5^Medical Association of Upper Austria, Quality Management, Linz, Austria; ^6^Department of Internal Medicine, Clinical Division of Internal Medicine II, Medical University of Innsbruck/Tirol Kliniken GmbH, Innsbruck, Austria; ^7^South Tyrol Rheumatology Unit, Hospital of Bruneck, Bruneck, Italy

**Keywords:** health care, workforce planning, career perspectives, rheumatology, educational initiative, medical training

## Abstract

**Objectives:**

A cumulative imbalance between rheumatologic need and an inadequate number of young colleagues entering the field leads to a dearth of rheumatologists in the near future. The Austrian Society for Rheumatology and Rehabilitation (ÖGR) has been organizing an annual Rheumatology Summer School (RSS) for medical students since 2017. The aim of this study was to analyze the annual RSS evaluations, the RSS’ overall effects on attracting new talent into the field and the lasting promotion of rheumatology.

**Methods:**

A questionnaire was distributed immediately after each RSS meeting. Additionally, we conducted an electronic survey among RSS participants (2017–2022) to assess their career development trajectories.

**Results:**

From 2017–2023, a total of 220 students attended the RSS. They all completed the annual evaluation. Accordingly, students’ expectations were met in 80% (2017) to 97% (2023) of cases. The electronic survey was completed by 64/133 (48%) students; 49 (77%) indicated that the RSS had markedly increased their desire to specialize in rheumatology. Among the 36 graduates, 10 (28%) had already been working in the field of rheumatology and 6 (17%) were considering this specialty but had not decided yet. RSS attendees in their 6th study year were influenced to a greater extent by the RSS to choose rheumatology as their primary specialty than 4th or 5th year students. The participants indicated that they benefited most from the RSS in terms of knowledge gain, personal awareness of rheumatology, networking among fellow students as well as gaining access to RSS faculty.

**Conclusion:**

The RSS enhanced students’ intention to choose rheumatology, particularly in those close to graduation, and led to increased awareness and deeper knowledge about rheumatology.

## 1 Introduction

After decades of consistently high numbers of graduates in the medical fields and an abundance of doctors, we are now facing an unprecedented lack of medical staff. In rheumatology, workforce studies have documented a current shortage of specialists in several countries, including Austria, and predicted a further increase of the gap between future demand and anticipated provision of qualified services (1–4). In general, strategies to increase the healthcare workforce have become an ever more popular topic (5, 6). Methods used to forecast these workforce requirements in the field of rheumatology are still quite heterogeneous, so the European umbrella organization for rheumatology, the European Alliance of Associations for Rheumatology (EULAR), provided guidance for future workforce studies, with the goal of reaching increased international comparability (7, 8).

Multiple factors contribute to the “slow leak” of rheumatology professionals: on the one hand, demographic developments within the population combined with increased frequency of rheumatic diseases in general, along with the growing complexity of medical care leads to a constantly increasing need for rheumatological services. On the other hand, specialists of the baby boomer generation are retiring, accompanied by a measurable decrease in new graduates entering rheumatology—caused in part by an increasing restriction of access to medical universities. Reformed European employment protections—in force in Austria since 2015—rigidly regulate working time to reduce the risk of physician burnout. Based on a considerable amount of hours spent outside the planned duty roster in the past, essential rota are not being covered any longer, and services are therefore lacking. Additionally, trends to work part- rather than full-time (not only among female professionals), alongside an increased desire to maintain a favorable work-life balance, further indicate a need for developing new talent (9–12). Since workforce gaps not only affect rheumatology but all medical disciplines and other professions in health care, competition for graduates to fill the gaps in the workforce of their particular field are intensifying.

Thus, a variety of education initiatives and training programs in medicine during summer or winter holidays have been developed in recent years, with the objective being to complement traditional academic curricula (13–19).

Some initiatives focus primarily on participants’ training in *academic* skills, and research in particular (e.g., communication and presentation skills for conference talks, research design, scientific writing etc.) (15–17). One of these (for addiction medicine) was evaluated over a period of twelve years in terms of early engagement of students and trainees in research and publication activities (17).

A project in oncoly has focused on improvements in clinical decision making, while another program in the same specialty aimed to expose students to specific clinical problems and to measure knowledge gain using pre- and post-course tests (18, 19). Another notable initiative is the summer school of the German Society for Rheumatology (Deutsche Gesellschaft für Rheumatologie, DGRh), held annually for an entire week in August since 2013. The DGRh summer school is open for students from the 4th year onwards, as. As with the RSS, the program includes theoretical and clinical content (20).

All these initiatives certainly have their merits. However, none of them evaluated the effects of the project on developing clinicians and academics in the relevant specialty. Still, the need for evaluation of educational activities is non-controversial (21, 22). Educational initiatives for young rheumatologists in training are provided by EULAR within a format of in-person international summer courses for various skills within the setting of the so-called EULAR school of rheumatology. Similarly, a European Summer School, organized by several cooperating national rheumatological societies, was in place from 2015 till 2017, also open only to trainees in rheumatology, not to students (23, 24).

At universities, the focus of the curriculum in medicine does not always correspond to the prevalence of diseases. Given that rheumatic diseases affect ~20% of the population, and musculoskeletal diseases’ profound impact on quality of life through chronic pain, possibly reduced mobility and productivity, along with social exclusion, rheumatology is clearly underrepresented at many universities (25, 26). Students in faculties of medicine are therefore often not aware of the content as well as career possibilities in rheumatology. Consequently, graduates tend to choose rheumatology less frequently than other fields of medicine in which students feel more experienced. To close this gap, the Austrian society for Rheumatology and Rehabilitation (Österreichische Gesellschaft für Rheumatologie, ÖGR) developed the Rheumatology Summer School (RSS), held annually since 2017. The RSS’ dates are always set directly following the summer semester’s annual examinations., in order not to interfere with clinical traineeships or summer jobs which students usually start later during their summer holidays. The RSS has undergone continuous development: currently, it is a 3 day course, composed of 20% theoretical lectures, 40% case-based learning and 40% practical skill sessions including musculoskeletal ultrasound, clinical examination, nailfold capillaroscopy, and synovial fluid analysis (see Supplementary material S1 for syllabus). Speakers and instructors—who volunteer their services—come from universities, community-based hospitals and private practices from all over Austria. In 2020, EULAR endorsed the RSS via its EULAR Educational Cooperation with National Societies (ECONS) program, and since then, EULAR has provided three international speakers each year.

The RSS is promoted via different channels: University offices, student representatives, social media, and former RSS-participants (via word of mouth). RSS participation is open to ~30 students per year and is budget-dependent. Applicants need to be studying medicine in a EULAR country, be in the 4th–6th year of the medical university curriculum and have sufficient language skills in German and English to follow the program successfully.

The RSS is free to participants; costs are covered by grants from pharmaceutical companies (multi-sponsoring), which have no influence on the program, promotion or invitation of students to the RSS.

The aim of this study was to investigate the annual evaluations from 2017–2023 and to analyze the overall effect of the RSS for students of medicine from 2017–2022 in terms of improving students’ overall perception and knowledge in rheumatology, as well as to boost the influx of students into the field of rheumatology as trainees, doctoral students or researchers.

## 2 Materials and methods

### 2.1 Evaluation procedures

#### 2.1.1 Annual evaluation

The evaluation was conducted anonymously immediately after the last RSS session each year. A paper-based questionnaire of 13 questions in a mixed format was used, including both multiple choice and open-ended questions (see Supplementary material S2 for full questionnaire). For verbal rating questions, Likert scales were applied (on a scale of 1 to 4 for most questions, leaving out the neutral middle answer in order to get explicitly positive or negative feedback) or, from 1 to 5 for the remainders, with 1 reflecting the best, 3 average or indifferent, and 5 the worst possible rating. The questionnaire was intended to reflect individual perceptions of the RSS’ content, e.g., the fulfillment of expectations, adequacy of RSS content, rating of practical skills sessions, knowledge gain, RSS organization, the effect of the RSS on individuals’ future career plans, and finally, if attendees would recommend the RSS to other students.

#### 2.1.2 Evaluation of career development of RSS participants from 2017 till 2022

A separate electronic survey was designed to investigate the effects of the RSS on the professional development of medical students covering the following topics (see Supplementary material S3 for full questionnaire):

- Demographic data
- Current working situation
- Further career plans in general
- Plans to specialize in rheumatology
- Academic activities in rheumatology (e.g., PhD thesis)
- Impact of the RSS on decisions concerning the individual career (specialization in rheumatology) as well as participation in further events and projects of the ÖGR

The questionnaire consisted of 13 questions and had a mixed format, containing multiple choice or open-ended questions as well as ratings using a 10-point numerical rating scale (NRS, ranging from 0–10, 0 = least and 10 = highest answer option).

When applying for participation in the RSS, students were asked whether they agreed to be contacted later by e-mail for questionnaires and other activities related to the evaluation of the RSS. From January to March 2023, the questionnaire was distributed electronically, using the survey tool “essentials Questback^®^.” After the first invitation, three email reminders were sent to non-responders. All participants gave informed consent to be contacted for evaluation procedures after the RSS. Participants could choose freely whether or not to complete the questionnaires.

### 2.2 Data analyses and statistics

For the statistical analyses, the statistic program ALMO 15 (developed and provided by the Johannes Kepler university Linz, Austria), applying interfaces with SPSS (SPSS Inc., IBM, Chicago, IL) was used. For demographic data of respondents as well as for explorative analysis of data, descriptive statistics were used providing the mean and standard deviation (SD) for numeric data as well as the number and proportions for categorical variables. *t*-tests and chi square tests were applied when appropriate. *p*-values <0.05 were considered statistically significant. Proportions have been calculated on the basis of the number of valid responses. Open questions were coded using a thematic approach.

## 3 Results

### 3.1 Annual evaluation of the rheumatology summer school 2017–2023

From 2017 to 2023, 220 students attended the RSS, comprising the following nationalities: Austria (*n* = 164), Germany (*n* = 40), Italy (*n* = 7), and one participant each from France, Georgia, Hungary, Poland, Russia, Spain, South Korea, Switzerland, and Syria (total *n* = 9). 209 students were studying at Austrian universities, the remaining 11 were studying abroad (8 in Germany, 1 in Slovakia, 1 in Spain, and 1 in Poland).

The number of RSS participants per year ranged from 28 (2019 and 2022) to 36 in 2018. The annual evaluation was completed by 100% of the RSS attendees, resulting in 220 responses in total.

**Figure 1:**
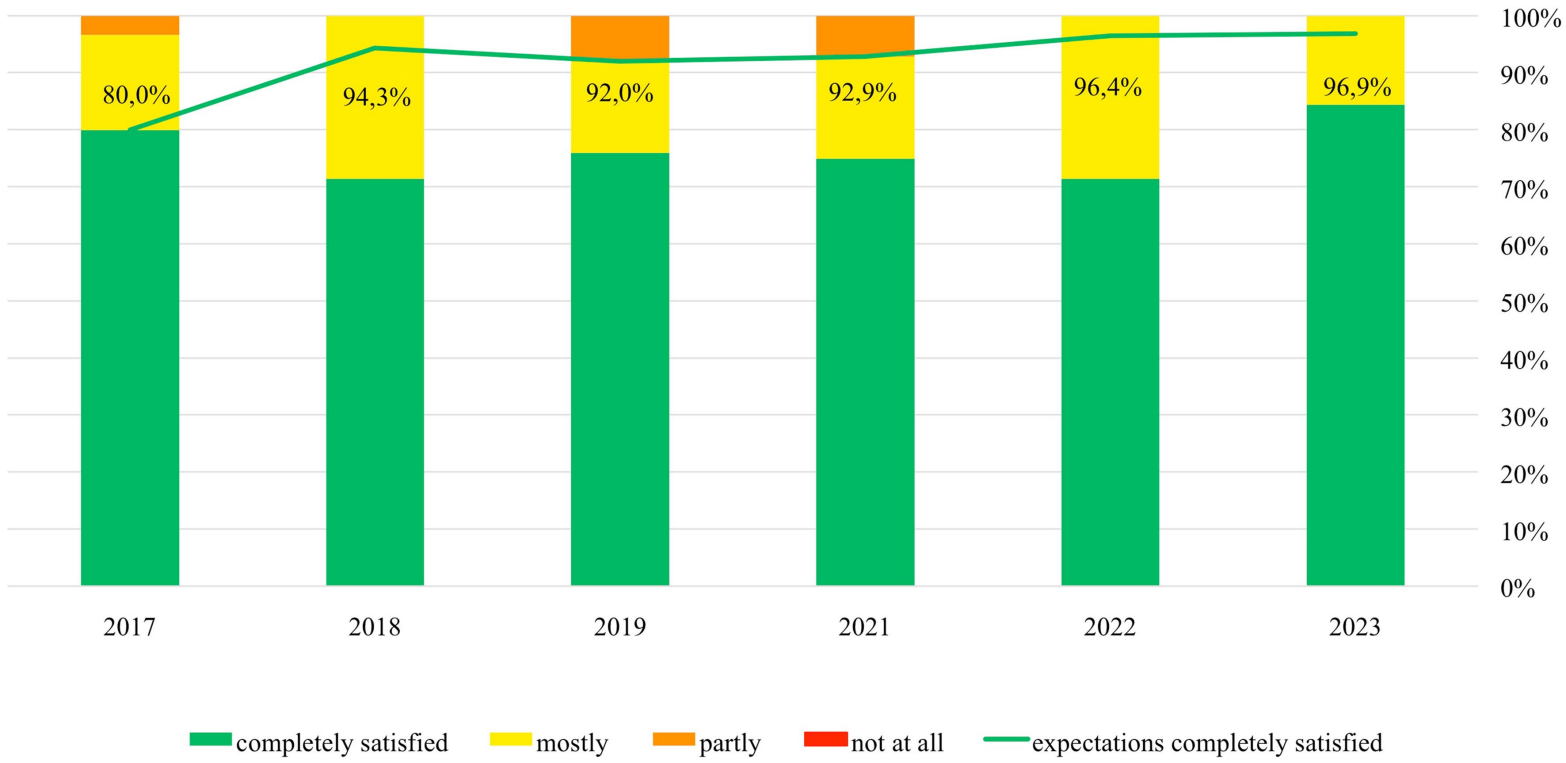
Annual evaluation of students’ satisfaction with their expectations in the Rheumatology Summer School (RSS) (green line) and students’ satisfaction with knowledge gain (bars) from 2017–2023 (2020 excluded, virtual format).

**Figure 2:**
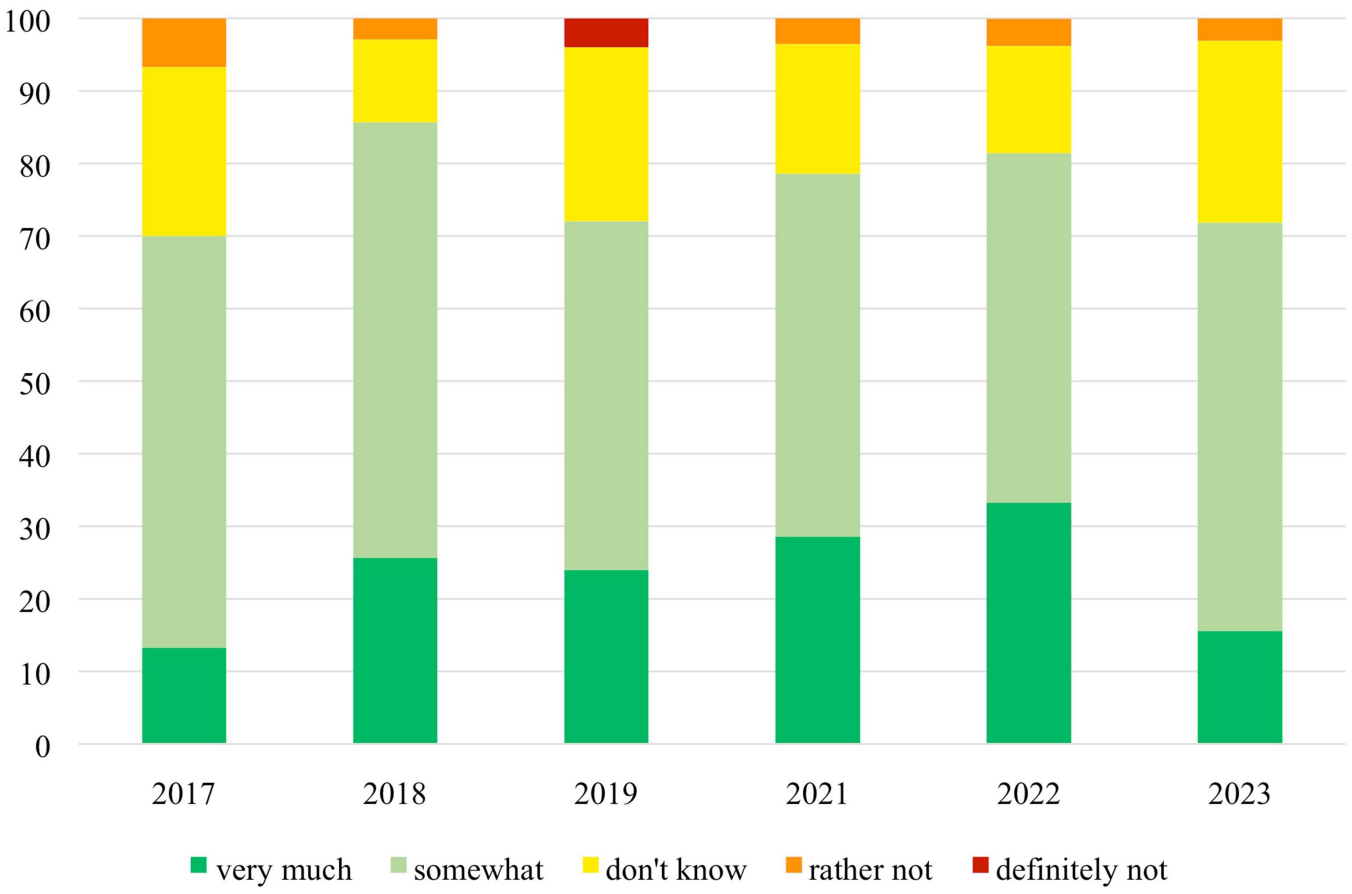
Annual evaluation of the impact of the Rheumatology Summer School (RSS) on participants’ desire to specialize in rheumatology per year (2020 excluded, virtual format).

From 2017 onwards, the percentage of students who stated that their expectations in the RSS were completely satisfied steadily increased from 80% in 2017 to 97% in 2023 (see Figure 1). In all editions of the RSS, more than 90% of participants indicated they had achieved significant knowledge gains. This number even increased over the years, being highest in 2023. The percentage of students rating the practical skills sessions with 1 (very good) continuously increased from 60% in 2017 to 91% in 2023. The ambition to specialize in rheumatology could be strengthened in >50% of participants every year (see Figure 2). All 220 participants responded that they would fully recommend the RSS to other students.

### 3.2 Evaluation of the rheumatology summer school professional outcome 2017–2022

We were able to reach 133/187 students attending the RSS from 2017–2022, having asked them to complete the electronic questionnaire. Given that the survey was carried out before the RSS 2023, no students from 2023 (*n* = 33) were included.

We were unable to contact 54 students (all of them participated in the RSS in 2017 or 2018) due to missing valid email accounts or current contact address/phone numbers.

Of the 133 students, 64 (48%) replied. The demographic data of the respondents are detailed in Table 1.

**Table 1:** Demographic data of all electronic survey respondents.

*N* (%) of RSS participants (2017–2022), holding a valid email account in 2023		187 (100%)
Gender	Female	109 (59%)
	Male	77 (41%)
	Diverse	1
Electronic survey addressees		133
*N* (%) of survey respondents		64 (48%)
Age (years, median)		24.1
*N* (%) of respondents per year of RSS attendance	2017	5 (7.8%)
	2018	12 (18.8%)
	2019	5 (7.8%)
	2020 (virtual format)	5 (7.8%)
	2021	19 (29.7%)
	2022	18 (28.1%)
Study year of RSS attendance	4th year	16 (25.0%)
	5th year	19 (29.7%)
	6th year	23 (35.9%)
	No answer	6 (9.4%)
Medical University of graduates (*n* = 36)	Med. Univ. Vienna	15
	Med. Univ. Graz	12
	Med. Univ. Innsbruck	6
	Med. Univ. Charité, Berlin	2
	Med. Univ. Regensburg	1

Students who attended the RSS in their 6th year represented the highest response rate (36%), followed by those in the 5th (30%) and in the 4th year (25%). Six participants did not respond to the question asking in which study year they attended the RSS.

### 3.3 Current status of RSS participants’ medical training

**Figure 3:**
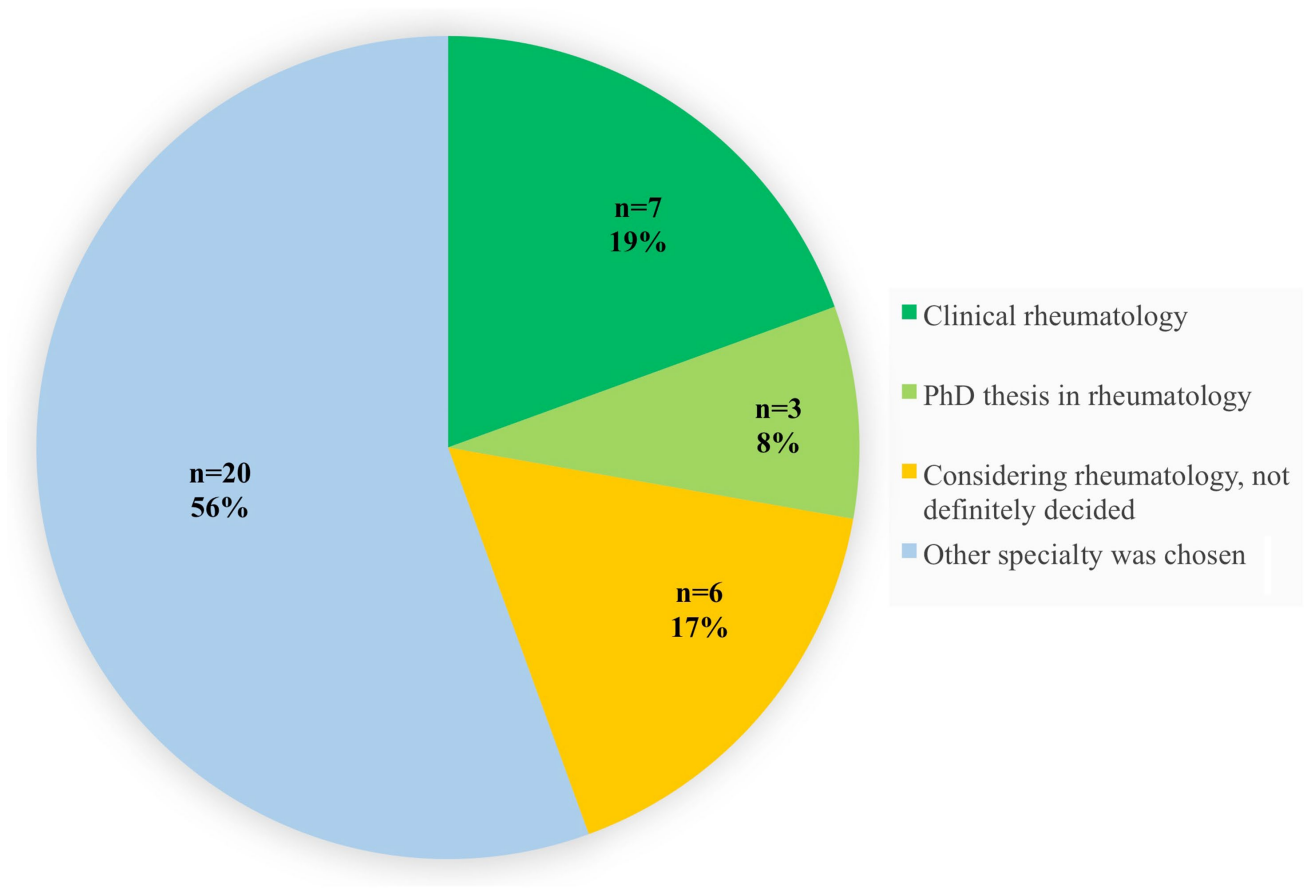
Career development of the 36 already graduated Rheumatology Summer School (RSS) participants (electronic survey).

At the time of the survey, 36 participants (56%) had already graduated, the other 28 (44%) had not yet finished their medical studies. Among the 36 graduates, 10 (28%) were working in the field of rheumatology: One of these was already a specialist, 6 were at different stages of their rheumatological training and 3 were completing a PhD thesis in rheumatology. Another six participants (17%) in training considered specializing in rheumatology but had not yet decided (see Figure 3). Twenty graduates (56%) chose other specialties.

Thirty-five of the 36 graduates were working as physicians and one was in the private sector (laboratory diagnostics).

### 3.4 Influence of the RSS on the participants’ specialization preference

All electronic survey respondents (*n* = 64) were asked whether the RSS changed their desire to specialize in rheumatology (see Figure 2; Supplementary material). In 49 (77%) respondents, the RSS had increased this desire with a mean increase of 2.7 ± 1.3 points on the NRS, from 4.7 ± 2.2 to 7.4 ± 1, possible range 0–10. Twelve (19%) stated that their desire had not changed, and three (4%) indicated a reduced interest in rheumatology after the RSS.

The increase in the desire to specialize in rheumatology after the RSS seemed to depend on the study year during which the students participated in the RSS. A higher increase on the NRS was detected in students from the 6th study year, as compared to those from the 4th and 5th year, as detailed in Table 2. In the sub-analysis of the 31 graduates with available data on the study year of RSS participation, we observed a statistically significant increase only in students from the 5th and 6th year, but not in those from year 4. Any direct comparisons between annual evaluations and responses to the electronic survey were not possible because the former were anonymous and could therefore not be assigned to an individual participant.

**Table 2:** Influence of the Rheumatology Summer School (RSS) on the desire to specialize in rheumatology in electronic survey respondents, measured on a 10 points numerical rating scale (NRS, 0–10, 0 = no interest in specializing, 10 = very high interest in specializing), graduates = at the time of the survey already graduated participants.

	Desire to specialize in rheumatology (NRS, 0–10)			*p*-value
	Before RSS	After RSS	Difference	
All students (*n* = 64)	5.3 (2.4)	7.3 (2.1)	2.0 (1.9)	***p* < 0.001**
Students specifying their study year (*n* = 58)				
4th study year (*n* = 16)	4.6 (2.1)	6.5 (1.7)	1.9 (1.8)	***p* < 0.001**
5th study year (*n* = 19)	5.7 (2.5)	7.4 (1.8)	1.6 (2.3)	***p* < 0.01**
6th study year (*n* = 23)	5.1 (1.9)	7.4 (2.2)	2.3 (1.5)	***p* < 0.001**
Graduates (*n* = 31)				
4th study year (*n* = 4)	4.8 (2.9)	6.3 (0.8)	1.5 (2.3)	*p* = 0.34
5th study year (*n* = 7)	4.6 (2.7)	7.0 (0.8)	2.4 (2.0)	***p* = 0.02**
6th study year (*n* = 20)	5.1 (1.9)	7.4 (2.2)	2.4 (1.7)	***p* < 0.001**

For statistical evaluation of the effect of the RSS, student’s *t*-test was applied with significant *p*-values (*p* < 0.05) highlighted in bold letters.

### 3.5 General impact of the RSS on the participants

Fifty-seven students (89%) answered that the RSS gave them new insights into rheumatology, while 53 (83%) responded that their knowledge about rheumatic diseases had improved significantly as a result of their participation. Awareness of rheumatology and rehabilitation as a specialty improved in 52 (81%) students; 51 (80%) reported to have established contacts with students from other universities and have started networking activities. All questions were answered dichotomously.

6 of the 36 graduates (17%) also participated in the RSS programme for medical trainees (available since 2021); two of these even advanced to the organizing committee for this event.

From 2017–2023, 70 of the 220 students (32%) attended the annual scientific meeting of ÖGR in the year of their RSS participation.

## 4 Discussion

In this study, we report the results of an innovative project in medical education, the ÖGR RSS, which has thus far hosted 220 students of medicine from twelve countries. We show that the vast majority of students experienced significant knowledge gains, influencing their overall perception of rheumatology. The RSS was able to increase students’ desire to specialize in rheumatology, and ~30% of graduates chose rheumatology either as a clinical specialty or for research.

To the best of our knowledge, this is the first study evaluating the effects of a summer school targeting fresh talent to consider entering the field of clinical and academic rheumatology.

### 4.1 General aspects of this summer school initiative

Trying to enhance the yield in new talent is in line with EULAR recommendations on workforce planning in rheumatology, underscoring the need that every effort should be made to increase the interest of graduates in rheumatology in order to ensure future workforce participation in the field (7, 8, 12, 25). In light of the demographically foreseeable lack of rheumatologists, all “marketing activities” for rheumatology, including the organization of summer schools and other events, can be regarded as a reasonable investment in the future.

The question of whether the graduates chose rheumatology because of their participation in the RSS or whether they had pre-selected this specialty already and the RSS merely reinforced their decision-making, is difficult to answer. Similarly, it is almost impossible to assess the effects of the RSS on the overall number of graduates entering the field of rheumatology in Austria. The evaluation of an educational project like the RSS cannot be designed like a RCT with a “control” or “placebo” group. In addition, there are several factors such as income, role models, personal experiences, job offers and associated duties of a rheumatologist, e.g., night shifts, etc., that might all influence the overall trends of graduates in medicine to choose one or the other specialty. This can neither be measured nor accounted for by statistical means. The main objective of the RSS was to promote rheumatology as a medical specialty and to fill possible knowledge gaps among students, given that rheumatology is underrepresented in most academic curricula in European countries. As with promotions of any kind, secondary effects—such as recommending this specialty to other students (word of mouth) or simply changing the perception of rheumatology on a broader level—are desirable, yet these effects are difficult to quantify.

### 4.2 Student feedback and continuous evolution of the RSS

Most students delivered a positive annual evaluation of the RSS from the start in 2017, and responses improved continuously over the years. We assume this was because of constant progress in the development of the RSS structure, based on student feedback, towards the incorporation of more practical skill sessions and case-based learning workshops as well as the integration of new formats like the Rheumatology Escape room or scientific walks. As already shown for a summer school project in oncology, it is crucial to improve the format of the event over time, to modernize it and render it more practical. The positive effect of this evolution was documented by ever-increasing student satisfaction with the summer school (27). Positive feedback from students alone, however, does not necessarily lead to an increase in younger colleagues entering the field. A summer school initiative for genomics and genetics, for example, reported that students were either very satisfied or satisfied with the project, however, no increase in the number of students entering residencies in genetics and genomics was subsequently observed (13).

Another important, yet difficult to measure effect, is the RSS as a platform for networking, exchanging ideas and finding a mentor within the ÖGR career track. By connecting with rheumatologists from across all Austrian medical universities as well as colleagues working in private practice, students of medicine were able, in an informal way, to learn more about career opportunities and job models in various fields, including work perspectives abroad, given the involvement of speakers from several EULAR countries. This closes the circle from being theoretically attracted by a sub-specialty to actually considering future job options. The development of mentor/mentee relationships is essential, particularly if they potentially last and become beneficial for further career development, also in the long term (28).

### 4.3 Strengths of the study

Our study has considerable strengths. It surveys a time period of 6 years—long enough to draw definite conclusions about the choices made by upcoming rheumatology clinicians and academics in Austria. No other summer school evaluation has covered such a long time period. The German Society of Orthopaedics and Trauma Surgery has published an evaluation of a summer school program overlooking a time period of just 5 years, yielding an even more pronounced positive effect on the increase in available clinical talent (29).

Another strength is that we have two evaluations of the RSS: the annual evaluation immediately after each summer school, being completed by 100% of the participants, and the electronic survey regarding RSS impact on career development. Both evaluations are consistent in the positive feedback of students, and they documented a statistically significant increase in students’ desire to specialize in rheumatology, particularly in those who were close to graduation.

Another aspect that can be seen as an effect of the RSS, is that, through the years, some RSS participants have been involved in special interest groups of the ÖGR such as the social media and visibility group or the Female Advancement In Rheumatology (FAIR) taskforce. In addition, the ÖGR board decided to create a new taskforce for young rheumatologists in Austria, called the young ÖGR (JÖGR), involving some former RSS participants. In 2021, the ÖGR expanded the summer school project to a full career track, where the RSS for students of medicine represents part 1, a summer school for residents/trainees in rheumatology part 2, and a rheumatology mentor-mentee program part 3.

### 4.4 Limitations of the study

Our study has some potential limitations. The number of respondents is small; however, we could only reach 48% of former RSS participants because of their restricted email availability. We asked students for their current email address when they participated in the RSS, which was mostly their university account, often terminated once they complete their medical studies. We could have asked for a private email address; however, there was no guarantee that such an email address was still in use some years after the RSS. The response rate of those who could be reached was higher than in other student surveys (30–33). Even a recent survey on educational topics conducted in Japan, a traditionally “hierarchically obedient” society, only yielded a response rate of 35% (34). In contrast to other educational programs, we did not conduct knowledge tests in the RSS, rather we asked for students’ perception of knowledge gain, which can be regarded a limitation of our project (18, 19).

In addition, Austria is a small country with only few rheumatology departments, allowing us to easily track more recent graduates who made their way into clinical or academic rheumatology in Austria. New colleagues who slipped under our radar must have either gone into another field of medicine or moved abroad. Another important limitation is that we may have had a bias toward the selection of highly motivated students, and that the effect could have been lower if students with no strong feelings for rheumatology had participated. Concerning the positive evaluation of the RSS, one could argue that students being invited to a summer school rate this experience well according to (unmeasurable) complimentary reasons, leading to bias. However, other scientific societies representing different sub-specialties in Austria, like, e.g., gastroenterology, facing the same lack of new talent, have initiated summer schools which emulate our model. This constitutes further competition for our RSS and may likely increase critical feedback. Moreover, attendee’s participation in further ÖGR activities like the RSS for medical trainees, the career track or the ÖGR annual scientific meeting is further proof of enduring interest.

## 5 Conclusion

Our study is the first to evaluate a national scientific society’s efforts to solve recruiting problems in rheumatology in the long term. It could demonstrate the RSS’ measurable positive impact on medical students’ future career decisions, thereby fostering young talent needed for clinical and academic rheumatology. The RSS strengthens the decision to specialize in rheumatology, especially in students nearing graduation. The participants positively rate the RSS in terms of knowledge gain, improved awareness of rheumatology in general, and networking opportunities. It also promoted and enabled the participation of young colleagues in the national scientific society. Further studies evaluating longer time periods and actual workforce developments are warranted.

## Data Availability

The raw data supporting the conclusions of this article will be made available by the authors, without undue reservation.
