# Supplementary material for: Professional development is the key to securing a future rheumatology workforce. Long term evaluation of a summer school for medical students—a national scientific society’s educational initiative
Source: Front Med (Lausanne). 2024 Sep 3;11:1413544. doi: 10.3389/fmed.2024.1413544 (PMC11409003; doi:10.3389/fmed.2024.1413544)
Supplement: Supplementary file 1 [file Data_Sheet_1.PDF]

Supplementary material:

File 1 (supplementary material): ÖGR – Rheumatology Summer School (RSS) – syllabus

Day 1

From 3 pm	Individual arrival
7:30 pm	Get-together, Welcome from the scientific organizing committee

Day 2

8:15 am	Welcome from the president of the Austrian Society for Rheumatology and Rehabilitation (ÖGR)
8:20 am	The European perspective of rheumatology: What does EULAR stand for?
8:30 am	Introductory lesson: How to approach rheumatic diseases
9:00 am	Case based learning (workshop in small groups):
	1) How to approach a patient who has joint pain.
	2) My patient has persistent fever!
	3) Chronic back pain is not always a disc problem.
	4) How to perform a check up on elderly patients with rheumatism.
	5) White fingers and strange skin lesions
10:00 am	Gout and other crystal arthropathies
10:20 am	Coffee break
10:30 am	Practical skills session I (workshop in small groups):
	1) Clinical examination in rheumatology (patients)
	2) “RheumEscape” (Escape room in rheumatology)
	3) X-ray and MRI reading
	4) Musculoskeletal ultrasound
	5) Microscopy of synovial fluids / nailfold capillaroscopy
12:00	Lunch break
1:00 pm	What is wrong with my back? Clinical presentation and diagnosis of spondylarthritis
1:25 pm	A life in pain always and everywhere - Fibromyalgia
1:50 pm	Case based learning (small groups, rotating)
2:50 pm	Practical skills session II (workshop in small groups, rotating)

4:20 pm	Professors' anecdotes: Why did I become a rheumatologist, and would I make the same choice again? (all faculty day 1)
5.00 pm	Self-guided learning in small groups

Day 2

9:00 am	Small vessel vasculitis, autoinflammation and rare diseases – challenging the rheumatologist
9:30 am	Case based learning (workshop in small groups, rotating)
10:30 am	Coffee break
10:45 am	*"The scales fell from my… joints"* – psoriatic arthritis
11.15 am	Case based learning (workshop in small groups, rotating)
12:15	Lunch break
1:15 pm	Case based learning (workshop in small groups, rotating)
2:15 pm	Practical skills session III (workshop in small groups, rotating)
3:45 pm	Scientific walk

Day 3:

<u>Block: Career and Science in Rheumatology</u>

9:00 am	Science made in Austria
9:20 am	Career as a scientist (in rheumatology)
9:40 am	Rheumatology training curricula, specialist exam and private practice
9:55 am	Why become a rheumatologist? Real life career experiences of a former RSS student
10:10 am	Part time working, family and career perspectives, ÖGR career track
10:30 am	Coffee break
11:00 am	Practical skills session IV (workshop in small groups, rotating)
12:30	Lunch break
1:30 pm	Practical skills session V (workshop in small groups, rotating)
3:00 pm	Feedback, evaluation, and concluding remarks from the scientific organizing committee

File 2 (supplementary material):

Questionnaire on the annual Rheumatology Summer School (RSS)–evaluation (paper based)

1. Have your expectations regarding the RSS been fulfilled?
   - o 1...completely
   - o 2...mostly
   - o 3...partly
   - o 4...not at all

   If your answer was <u>not</u> 1 (completely), please tell us why:

2. How satisfied are you with your knowledge gain in rheumatology?
   - o 1…completely
   - o 2...mostly
   - o 3...partly
   - o 4...not at all

   If your answer was <u>not</u> 1 (completely), please tell us why:

3. Did you feel challenged enough or did you feel overstrained by the RSS?
   - o Neither / nor
   - o Overstrained because:
     - o too fast; I could not follow the curriculum
     - o the learning objectives were too difficult
     - o other reasons
   - o Not challenged enough because:
     - o too slow, it was boring for me
     - o the learning objectives were too easy
     - o the content was already well known
4. Please rate the RSS using school marks (1=best, 5=worst):

	1	2	3	4	5
Lectures were interesting					
Schedule was reasonable					
Speakers were competent					
Speakers addressed relevant issues					

5. How do you rate the practical skills sessions (using school marks, 1=best, 5=worst)?

o 1 o 2 o 3 o 4 o 5

6. What did you like the most at the RSS?

(free text answer)

7. Was there anything you did not you like about the RSS? What would you do differently / change, being a RSS-speaker/faculty?

   (free text answer)

8. Did the RSS contribute positively to your desire to specialize in rheumatology?
   - o Yes, very much, I would like to specialize in rheumatology
   - o Somewhat
   - o Neutral / I can't tell at the moment
   - o Hardly
   - o Definitely not

9. How do you rate the organization of the RSS (using school marks, 1=best, 5=worst)?

   o 1 o 2 o 3 o 4 o 5

10. How do you rate the RSS venue (using school marks, 1=best, 5=worst)?

    o 1 o 2 o 3 o 4 o 5

11. Generally, did you feel comfortable at the RSS?

    o Yes o quite ok o no

12. How do you rate the RSS in general (using school marks, 1=best, 5=worst)?

    o 1 o 2 o 3 o 4 o 5

13. Would you recommend other students to attend the RSS?

o definitely o maybe o I don't know o probably not o definitely not

File 3 (supplementary material): Questionnaire on the professional outcome of the Rheumatology Summer School (RSS), for participants from 2017-2022 (electronic survey)

1. If you have already graduated, in which year?
   - 2016
   - 2017
   - 2018
   - 2019
   - 2020
   - 2021
   - 2022
   - I have not yet graduated
2. From which university did you graduate?
   - Medical University of Vienna (MUW)
   - Medical University of Graz
   - Medical University of Innsbruck
   - Kepler University Linz
   - Paracelsus Private Medical University Salzburg
   - Karl Landsteiner Private Medical University, Krems
   - Other: ………………………………
3. Your current field of activity:
   - Specialist in rheumatology
   - In training in rheumatology
   - In training in internal medicine, subsequently rheumatology
   - In training in internal medicine, heading for another subspecialty
   - Common trunk training, intention to specialize in rheumatology later
   - Common trunk training, considering specialization in rheumatology
   - Common trunk training, intention to specialize in a different field
   - PhD studies
   - Student
   - In training in another specialty ……………………………………………..
4. At which department are you working at present? (voluntary question)

5. In which study year did you attend the RSS?
   - 4th year

- o 5th year
- o 6th year
- o Don't know

6. Desire to specialize in rheumatology BEFORE the RSS

0 1 2 3 4 5 6 7 8 9 10

no interest very high interest

7. Desire to specialize in rheumatology AFTER the RSS

0 1 2 3 4 5 6 7 8 9 10

no interest very high interest

8. The RSS has *(tick all boxes that apply)*
   - o increased my personal awareness for rheumatology
   - o enabled insights into rheumatology, I would have been unable to get without it
   - o markedly increased my knowledge about rheumatology
   - o enabled networking in the rheumatology community
   - o enabled contact with a rheumatology university clinic
   - o enabled networking with students from other universities
   - o had a lasting effect on my personal approach to rheumatologic patients
   - o not influenced my later professional life
   - o showed me that rheumatology is not the field in which I want to specialize

9. If you have already started your training in rheumatology: Did the RSS influence this decision?

0 1 2 3 4 5 6 7 8 9 10

not at all very much

10. Have you finished a PhD-thesis, or are you currently working on it?
    - o Yes o No
11. If you have answered question 10 with yes:

    Specialty of thesis:

    University:
12. Did you attend the ÖGR annual scientific meeting in the year you were attending the RSS?
    - o Yes o No
13. Did you attend the RSS for medical trainees following the RSS for medical students?
    - o Yes, year of attendance: o No

Demographic data

Year of birth

Gender

Year you started studying medicine

Name (optional)
